# Prognostic value of C-reactive protein and neutrophil-to-lymphocyte ratio in patients with hepatocellular carcinoma

**DOI:** 10.1186/1471-2407-13-78

**Published:** 2013-02-15

**Authors:** Byong Sun Oh, Jeong Won Jang, Jung Hyun Kwon, Chan Ran You, Kyu Won Chung, Chul Seung Kay, Hyun Suk Jung, Seungok Lee

**Affiliations:** 1 Department of Internal Medicine, The Catholic University of Korea, Incheon St. Mary’s Hospital, Incheon, Korea; 2 Department of Radiation Oncology, The Catholic University of Korea, Incheon St. Mary’s Hospital, Incheon, Korea; 3 Department of Radiology, The Catholic University of Korea, Incheon St. Mary’s Hospital, Incheon, Korea; 4 Department of Laboratory Medicine, The Catholic University of Korea, Incheon St. Mary’s Hospital, Incheon, Korea; 5 Division of Hepatology, Department of Internal Medicine, College of Medicine, The Catholic University of Korea, #222 Banpo-daero 22-gil, Seocho-gu, Seoul, 137-701, Korea

**Keywords:** Inflammation markers, Neutrophil-to-lymphocyte ratio, C-reactive protein, Hepatocellular carcinoma, Survival

## Abstract

**Background:**

Accumulating evidence indicates that components of the systemic inflammatory response, such as C-reactive protein (CRP) and neutrophil-to-lymphocyte ratio (NLR), have been associated with prognosis of various cancers. We aimed to elucidate whether CRP and NLR could serve as potential surrogate markers for response and survival in patients with hepatocellular carcinoma (HCC).

**Methods:**

The study population consisted of 318 consecutive patients with HCC. CRP and NLR were measured at baseline with follow-up measurements.

**Results:**

With the mean follow-up of 13.9 months, the median survival time was 13.8 months. Child-Pugh class, tumor size > 5 cm, tumor multiplicity, presence of portal vein thrombosis, α-fetoprotein > 200 ng/mL, CRP > 6.3 mg/L and NLR > 2.3 were identified as independent factors for worse survival of HCC (all *p* < 0.05). Patients with elevated CRP (> 6.3 mg/L) and elevated NLR (> 2.3) had a significantly shorter overall survival than those with low CRP and low NLR (all *p* < 0.001). The combined use of CRP and NLR provided incremental prognostic information. With significant inter-correlations, levels of CRP and NLR escalated with aggravating Child-Pugh class from A to C or progressing tumor stage from I to IV. CRP and NLR on baseline and serial measurements were well predictive of treatment response (*p* < 0.001).

**Conclusions:**

CRP and NLR are independent indicators for survival in HCC patients, reflecting tumor burden and hepatic reserve. Their role in predicting tumor response and survival is more enhanced when used in combination. This study suggests that CRP and NLR are important prognostic biomarkers for HCC.

## Background

Hepatocellular carcinoma (HCC) is the fifth most common malignancy and the third leading cause of cancer-related deaths worldwide [1]. Although there have been many advances in the treatment of HCC, the outcome is still not satisfactory [1,2]. Since a long lasting inflammatory process like cirrhosis continually induces hepatocarcinogenesis, there are limitations on curative therapy. Besides known prognostic factors representing tumor status and liver function [3], it is now clear that inflammation plays a significant role in tumor progression [4]. In this situation, C-reactive protein (CRP) and neutrophil-to-lymphocyte ratio (NLR), indicators of inflammation, have been suggested as surrogate markers for a relationship between inflammation and cancer [5].

CRP has been identified as a prognostic factor for HCC [6-10] as well as other various malignancies, such as gastrointestinal tumor, renal cell cancer and ovarian cancer [5,11]. It is an acute phase reactant, synthesized in the liver [12], and regulated by proinflammatory cytokines, like interleukin (IL)-6, which plays an important role in carcinogenesis [13]. The NLR has recently been evaluated as a predictor of prognosis of HCC [14-16] as well as other malignancies like colorectal cancer, gastric cancer, breast cancer and ovarian cancer [5,17-20]. It has been shown that high levels of NLR could predict a risk of recurrence and survival in patients with various malignancies.

HCC is unique among other cancers, in that its prognosis not only depends on the tumor characteristics but also on the hepatic functional reserve. In this respect, it seems to be more relevant to understand inflammatory process in hepatocarcinogenesis and evaluate its effect on the patient prognosis. To date, there have been no comprehensive data on the relationship between the inflammatory markers and hepatic reserve and tumor status in HCC. In addition, the significance of both CRP and NLR in HCC survival has not yet been explicitly studied in one research.

In the present study, we therefore evaluated the clinical value of CRP as well as NLR measured at the same point in time in clinical outcome in a large number of patients with HCC. In addition, changes in CRP and NLR after treatment were examined in relation to the treatment response. The present findings show the prognostic utility of both CRP and NLR as a surrogate marker for efficacy in treatment as well as HCC survival.

## Methods

### Patients

A database of all 318 patients with newly diagnosed HCC between January 2007 and December 2010 at Incheon St. Mary’s hospital, Incheon, South Korea was prospectively collected and retrospectively analyzed. The diagnosis of HCC was made based on histological evidence or elevated serum α-fetoprotein (AFP) levels (> 200 ng/mL) with typical radiological findings (arterial enhancement with portal washout) [21]. The staging of tumor was determined according to the TNM classification of Malignant Tumors/International Union Against Cancer (UICC) classification system, which is widely used in Korea [21]. Our main treatment modality for HCC was transarterial chemotherapy (TAC)-based loco-regional therapy. The chemotherapeutic regimen used for TAC was intra-arterial chemotherapy using doxorubicin (50 mg) for patients who had multifocal tumors ≤ 10 cm or a combination of epirubicin (50 mg) and cisplatin (60 mg) for patients with Child-Pugh class A who had large tumors (> 10 cm) with or without portal vein thrombosis (PVT). Patients with PVT or extrahepatic metastasis were considered for radiotherapy in addition to TAC. No patients treated with loco-regional therapy were given glucocorticoids. Each patient provided informed consent to participate in the study. This study was approved by the Ethics Committees of The Catholic University of Korea in accordance with the 1975 Declaration of Helsinki.

### Methods

Laboratory data including CRP and NLR were obtained from the study subjects prior to the initiation of treatment and at each treatment cycle. All serum samples were measured as fresh state. Serum CRP level was determined as highly-sensitive CRP (hs-CRP) by immunoturbidimetric assay using CRPH (C-Reactive Protein, High Sensitivity) reagent (Beckman Coulter, Inc., Fullerton, CA USA; limit of detection, 0.08 mg/L). The NLR was calculated by dividing the neutrophil count by the lymphocyte count. CRP and NLR measurements were obtained without demonstrable infection. To evaluate the relationship between changes in CRP levels and NLR and tumor response, serial measurements of the CRP levels and NLR were carried out 3 to 4 months after treatment. Based on the modified Response Evaluation Criteria In Solid Tumors (RECIST) criteria [22], patients with complete response and partial response after treatment were grouped together as responders, whereas patients with stable disease and progressive disease were defined as non-responders.

### Statistical analysis

SPSS version 18 (SPSS Inc., Chicago, IL, USA) and MedCalc version 11.6.1 (MedCalc Software Inc., Mariakerke, Belgium) were used to analyze the data. The cut-off values of CRP and NLR were determined using receiver operating characteristic (ROC) curve analysis. The optimal cut-off levels for CRP and NLR were established at 6.3 mg/L and 2.3, respectively, and these cutoff values were used to categorize the high and low CRP or NLR groups. Univariate analysis was performed to assess significant differences in clinical characteristics. A multivariate analysis was performed by Cox regression for variables significant on univariate analysis. Overall survival was calculated from the date of diagnosis to the date of death or last follow-up. For patients undergoing liver transplantation, follow-up was censored at the time of transplantation. To compare overall survival rates according to CRP or NLR, the data were analyzed by the Kaplan-Meier method, and the differences in survival between groups were compared by the log-rank test. Correlations between the inflammatory markers and Child-Pugh class and tumor stage were assessed using Spearman correlation coefficients. Serial changes in CRP levels and NLR were evaluated using repeated measures ANOVA. Each continuous variable was appropriately categorized in order to achieve the largest statistical power. A *p* value of less than 0.05 was considered to be statistically significant.

## Results

### Baseline characteristics

A total of 318 patients were consecutively enrolled in this study. Table 1 shows baseline characteristics of the entire study population. Patient age ranged from 32 to 89 years (median: 58 years) and 240 (75.5%) of the patients were males. At diagnosis, 200 (62.9%) patients were classified into Child-Pugh class A; 91 (28.6%) patients into Child-Pugh class B; 27 (8.5%) patients into Child-Pugh class C. The median tumor size was 5.5 (0.8–26.5) cm and the number of patients with solitary tumor was 144 (45.3%). Each case of presence of PVT and extrahepatic metastasis was 107 (33.6%) and 62 (19.5%), respectively. Distribution of tumor stage after UICC (I/II/III/IVa/IVb) classification in our patients was as follows: 41 (12.9%)/76 (23.8%)/85 (26.7%)/64 (20.1%)/52 (16.4%), respectively. The median levels of serum AFP, CRP and NLR were 92.3 (1.6–2,753,500) ng/mL, 4.7 (0.1–343.6) mg/L and 2.6 (0.6–49.9), respectively. The allocated treatments for the 318 patients were surgical resection (n = 41), liver transplantation (n = 10), TAC-based loco-regional therapy (n = 221), and supportive care (n = 46).

**Table 1:** Baseline patient characteristics according to total patients, CRP and NLR level

**Characteristics**	**Total patients (n = 318)**	**Patients grouped by CRP level (n = 318)**		**Patients grouped by NLR level (n = 318)**	
		**CRP ≤ 6.3 mg/L (n = 171)**	**CRP > 6.3 mg/L (n = 147)**	**NLR ≤ 2.3 (n = 129)**	**NLR > 2.3 (n = 189)**
Sex (male:female)	240:78	122:49	118:29	93:36	147:42
Age (years)	58 (32–89)	59 (33–80)	58 (32–89)	58 (33–80)	59 (32–89)
Cause (HBV/HCV/Alcohol/Others, %)	242 (76.1)/ 33 (10.4)/ 25 (7.9) / 18 (5.7)	123 (71.9)/ 26 (15.2)/ 12 (7.0)/ 10 (5.8)	119 (81.0)/ 7 (4.8)/ 13 (8.8)/ 8 (5.4)	100 (77.5)/ 14 (10.9)/ 8 (6.2)/ 7 (5.4)	142 (75.1)/ 19 (10.1)/ 17 (9.0)/ 11 (5.8)
Platelet counts (×10^3^/mm^3^)	139.6 ± 86.5	121.1 ± 63.9	161.1 ± 103.1	121.8 ± 64.9	151.7 ± 96.9
ALT (IU/L)	39.0 (1.0–2,430.0)	35.0 (1.0–2,430.0)	42.0 (6.0–503.0)	39.0 (1.0–2,430.0)	39.0 (6.0–503.0)
Total bilirubin (mg/dL)	1.0 (0.2–26.9)	0.9 (0.2–9.4)	1.2 (0.2–26.9)	0.9 (0.2–7.8)	1.1 (0.2–26.9)
Albumin (g/dL)	3.5 ± 0.6	3.7 ± 0.4	3.4 ± 0.5	3.6 ± 0.6	3.5 ± 0.6
Prothrombin time (%)	79.4 ± 18.0	81.3 ± 17.6	77.1 ± 18.2	80.4 ± 18.4	78.6 ± 17.7
Child-Pugh class (A/ B/ C, %)	200 (62.9)/ 91 (28.6)/ 27 (8.5)	128 (74.9)/ 36 (21.1)/ 7 (4.1)	72 (49.0)/ 55 (37.4)/ 20 (13.6)	99 (76.7)/ 26 (20.2)/ 4 (3.1)	101 (53.4)/ 65 (34.4)/ 23 (12.2)
Tumor size (cm)	5.5(0.8–26.5)	3.0 (0.8–18.6)	10.0 (1.0–26.5)	3.0 (0.8–17.4)	7.9 (1.3–26.5)
Tumor number, solitary (%)	144 (45.3)	93 (54.4)	51 (34.7)	74 (57.4)	70 (37.0)
Presence of PVT (%)	107 (33.6)	24 (14.0)	83 (56.5)	23 (17.8)	84 (44.4)
Presence of metastasis (%)	62 (19.5)	14 (8.2)	48 (32.7)	11 (8.5)	51 (27.0)
Tumor stage (I/ II/ III/ IVa/ IVb, %)	41 (12.9)/ 76 (23.8)/ 85 (26.7)/ 64 (20.1)/ 52 (16.4)	34 (19.9)/ 57 (33.3)/ 51 (29.8)/ 17 (9.9)/ 12 (7.0)	7 (4.8)/ 19 (12.9)/ 34 (23.1)/ 47 (32.0)/ 40 (27.2)	30 (23.3)/ 40 (31.0)/ 35 (27.1)/ 18 (14.0)/ 6 (4.7)	11 (5.8)/ 36 (19.0)/ 50 (26.5)/ 46 (24.3)/ 46 (24.3)
AFP (ng/mL)	92.3 (1.6–2,753,500.0)	28.9 (1.6–331,580.0)	350.0 (2.2–2,753,500.0)	32.3 (1.7–331,580.0)	207.1 (1.6–2,753,500.0)
CRP (mg/L)	4.7 (0.1–343.6)	1.2 (0.1–6.3)	24.9 (6.4–343.6)	1.7 (0.1–64.8)	14.3 (0.2–343.6)
NLR	2.6 (0.6–49.9)	1.8 (0.6–13.0)	4.0 (0.7–49.9)	1.5 (0.6–2.2)	3.8 (2.3–49.9)

*CRP*, C-reactive protein; *NLR*, neutrophil-to-lymphocyte ratio; *HBV*, hepatitis B virus; *HCV*, hepatitis C virus; *ALT*, alanine aminotransferase; *PVT*, portal vein thrombosis; *AFP*, α-fetoprotein.

### Factors affecting prognosis of HCC in the whole study population

To identify factors for HCC survival, 12 potential variables of interest were analyzed, as listed in Table 2. Of these, elevated ALT, Child-Pugh class, tumor size, tumor multiplicity, presence of PVT, presence of metastasis, elevated AFP, high CRP and high NLR were significantly associated with poorer survival. With multivariate analysis using a Cox regression model, Child-Pugh class (*p* < 0.001; Hazard ratio [HR] 1.711; 95% confidence interval [CI] 1.377–2.125), tumor size > 5 cm (*p* = 0.003; HR 1.778; 95% CI 1.209–2.615), tumor multiplicity (*p* = 0.035; HR 1.391; 95% CI 1.023–1.892), presence of PVT (*p* = 0.001; HR 1.827; 95% CI 1.284–2.598), AFP > 200 ng/mL (*p* = 0.001; HR 1.734; 95% CI 1.248–2.407), CRP > 6.3 mg/L (*p* = 0.027; HR 1.519; 95% CI 1.049–2.199) and NLR > 2.3 (*p* = 0.009; HR 1.601; 95% CI 1.124–2.280) were identified as independent poor prognostic factors for HCC (Table 2). When we included the combination of high CRP and high NLR as a variable into the analysis, the combination of CRP and NLR (*p* < 0.001; HR 1.905; 95% CI 1.345–2.697) together with Child-Pugh class (*p* < 0.001; HR 1.806; 95% CI 1.464–2.228), tumor size > 5 cm (*p* = 0.002; HR 1.858; 95% CI 1.258–2.743), presence of PVT (*p* < 0.001; HR 1.893; 95% CI 1.329–2.697) and AFP > 200 ng/mL (*p* < 0.001; HR 1.821; 95% CI 1.324–2.504) were identified as independent factors for worse survival instead of CRP or NLR alone.

**Table 2:** Univariate and multivariate analysis of prognostic factors of overall survival by Cox regression model

	**Univariate**		**Multivariate**	
	**HR (95% CI)**	***P *value**	**HR (95% CI)**	***P *value**
Sex (male)	0.842 (0.604–1.173)	0.309		
Age > 60 (years)	0.777 (0.586–1.031)	0.080		
Cause (viral)	0.978 (0.652–1.469)	0.916		
ALT > 40 (IU/L)	1.511 (1.146–1.992)	0.003	1.333 (0.985–1.804)	0.062
Child-Pugh class	1.803 (1.480–2.198)	< 0.001	1.711 (1.377–2.125)	< 0.001
Tumor size > 5 (cm)	3.548 (2.622–4.801)	< 0.001	1.778 (1.209–2.615)	0.003
Tumor multiplicity (≥ 2)	2.023 (1.510–2.711)	< 0.001	1.391 (1.023–1.892)	0.035
Presence of PVT	4.197 (3.141–5.609)	< 0.001	1.827 (1.284–2.598)	0.001
Presence of metastasis	3.002 (2.178–4.139)	< 0.001	1.242 (0.868–1.779)	0.236
AFP > 200 (ng/mL)	2.783 (2.099–3.690)	< 0.001	1.734 (1.248–2.407)	0.001
CRP > 6.3 (mg/L)	3.923 (2.929–5.255)	< 0.001	1.519 (1.049–2.199)	0.027
NLR > 2.3	3.050 (2.223–4.185)	< 0.001	1.601 (1.124–2.280)	0.009

*HR*, hazard ratio; *CI*, confidence interval; *ALT*, alanine aminotransferase; *PVT*, portal vein thrombosis; *AFP*, α-fetoprotein; *CRP*, C-reactive protein; *NLR*, neutrophil-to-lymphocyte ratio.

### Overall survival according to CRP and NLR level

**Figure 1:**
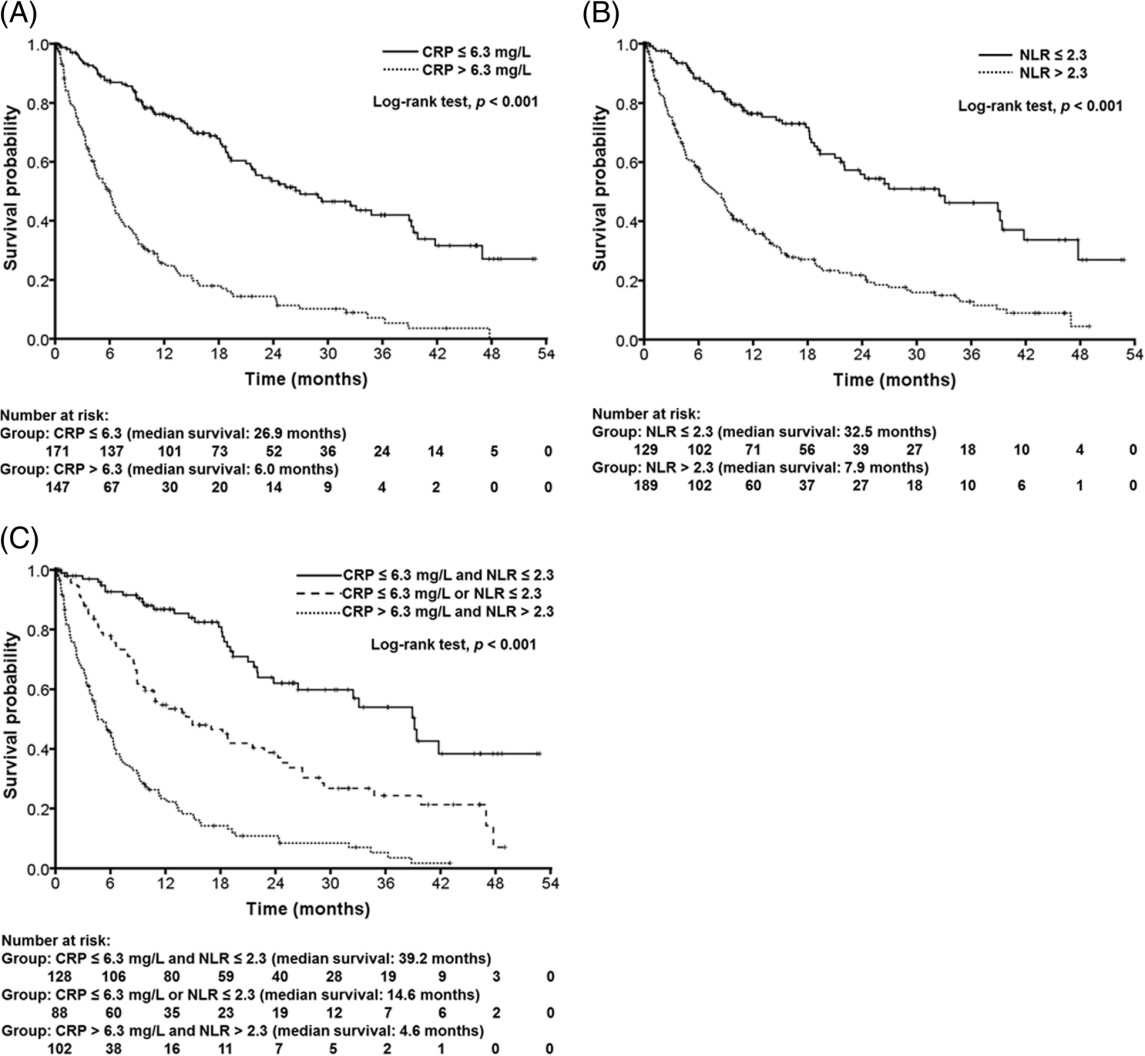
**Kaplan-Meier curves for overall survival probability according to (A) CRP, (B) NLR and (C) a combination of CRP and NLR. **(**A**) Patients with CRP > 6.3 mg/L (dotted line) had a significantly shorter overall survival than those with CRP ≤ 6.3 mg/L (solid line) (median 6.0 vs. 26.9 months, respectively; *p* < 0.001). (**B**) Likewise, survival among patients with NLR > 2.3 (dotted line) was shorter than those with NLR ≤ 2.3 (solid line) (median 7.9 vs. 32.5 months, respectively; *p* < 0.001). (**C**) There is a significantly longer survival in the group with both CRP ≤ 6.3 mg/L and NLR ≤ 2.3 (solid line, 39.2 months) than the group with either CRP > 6.3 mg/L or NLR > 2.3 (dashed line, 14.6 months) or than the group with both CRP > 6.3 mg/L and NLR > 2.3 (dotted line, 4.6 months).

During the mean follow-up period of 13.9 months, 202 (63.5%) of the patients died. The median survival time was 13.8 months. Since CRP and NLR played a significant role in predicting the HCC survival, we evaluated the differences in survival according to the low versus high CRP and NLR levels among the entire group. As depicted in Figure 1A, the median survival of patients with elevated CRP (> 6.3 mg/L) was 6.0 months, which was significantly shorter than 26.9 months for patients with a low level of CRP (log-rank test, *p* < 0.001). Likewise, the survival in the elevated NLR (> 2.3) group was significantly worse than that in the low NLR group, with the median survival times of 7.9 versus 32.5 months (log-rank test, *p* < 0.001; Figure 1B). The statistical differences for survival by the levels of CRP and NLR were still maintained in the patients receiving treatments as well as the entire patients (data not shown). An additional analysis was done to examine the synergistic effect of a combined use of CRP and NLR on the patient outcome. When the Kaplan-Meier survival curves were plotted over time, there was a stepwise increase in the overall survival rate from both high CRP (> 6.3 mg/L) and NLR (> 2.3) levels, one of them and to both low CRP (≤ 6.3 mg/L) and NLR (≤ 2.3) levels, indicating the benefit of the combined use of the two inflammatory markers (Figure 1C).

### Correlation of CRP and NLR with Child-Pugh class and tumor stage

**Figure 2:**
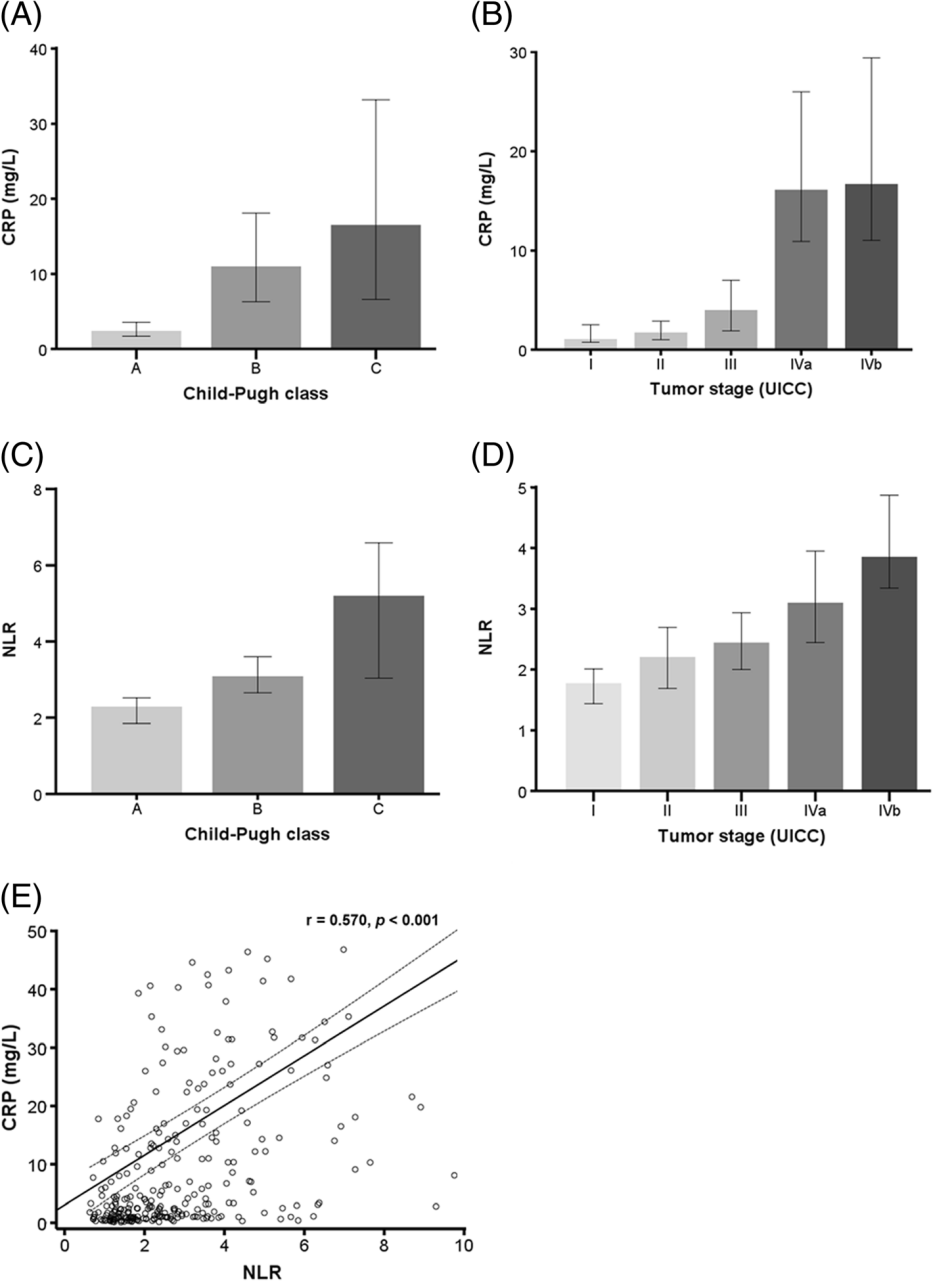
**Correlations between (A) CRP and Child-Pugh class, (B) CRP and tumor stage, (C) NLR and Child-Pugh class, (D) NLR and tumor stage, and (E) CRP and NLR.** The levels of CRP and NLR tends to escalate with aggravating Child-Pugh class from A to C (**A**, r = 0.311, *p* < 0.001; **C**, r = 0.306, *p* < 0.001) or progressing tumor stage from I to IV (**B**, r = 0.475, *p* < 0.001; **D**, r = 0.358, *p* < 0.001). (**E**) There is a significant positive inter-correlation between CRP and NLR (r = 0.570, *p* < 0.001).

The relationship of CRP and NLR with Child-Pugh class and tumor characteristics, which were the two major determinants of the prognosis of patients with HCC, were evaluated. There was a significant correlation between CRP concentrations and Child-Pugh class or tumor stage (r = 0.311, *p* < 0.001; r = 0.475, *p* < 0.001, respectively). As shown in Figures 2A and 2B, the level of CRP tended to increase as liver disease progressed from Child-Pugh class A to C as well as tumor stage from I to IV. Likewise, there was also a significant correlation between NLR and Child-Pugh class or tumor stage (r = 0.306, *p* < 0.001; r = 0.358, *p* < 0.001, respectively). Figure 2C and Figure 2D illustrate the escalating values of NLR with aggravating Child-Pugh class from A to C or progressing tumor stage from I to IV. When analyzed regarding the relationship between the CRP levels and NLR, there was a significant positive inter-correlation between CRP and NLR levels (r = 0.570, *p* < 0.001; Figure 2E).

### Tumor response according to CRP and NLR level

**Figure 3:**
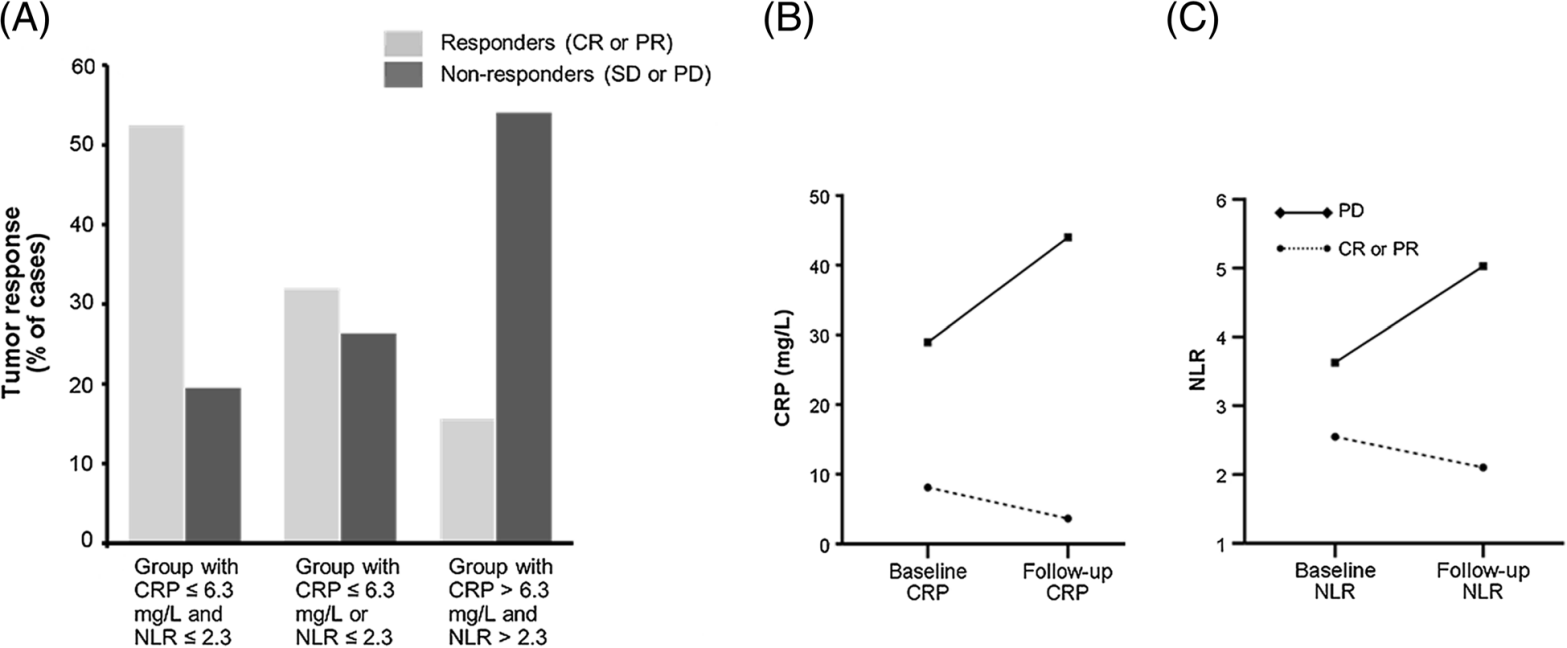
**Tumor response according to baseline levels of serum (A) CRP and NLR and changes in (B) CRP and (C) NLR levels after therapy. **Tumor response was better in the group with both baseline serum CRP ≤ 6.3 mg/L and NLR ≤ 2.3 than the other groups (p < 0.001; Figure 3A). At follow-up, patients with progressive disease showed an increase in levels of CRP and NLR, whereas responders exhibited a decrease in the levels (repeated measures ANOVA, all p < 0.001; Figure 3B and 3C). CR, complete response; PR, partial response; SD, stable disease; PD, progressive disease.

When baseline levels of CRP and NLR were analyzed for tumor response, there were more non-responders in the group with serum CRP > 6.3 mg/L and NLR > 2.3, whereas there were more good responders in the group with both baseline serum CRP ≤ 6.3 mg/L and NLR ≤ 2.3 (*p* < 0.001; Figure 3A). We next examined whether the changes in CRP concentrations or NLR serially measured before and after therapy could predict tumor response. At follow-up, compared with responders, patients with progressive disease showed an increase in levels of CRP and NLR. In contrast, responders exhibited a decrease in serial measurement of serum CRP and NLR (Figures 3B and 3C). There were statistically significant differences in the CRP and NLR changes between responders and those with progressive disease (all *p* < 0.001, repeated measures ANOVA).

## Discussion

The present study focused on the predictive value of CRP and NLR in the outcome of patients with HCC. From the analysis of a large cohort, we found that elevated CRP and NLR independently predicted worse survival in patients with HCC. In this study, CRP and NLR were utilized as prognostic indicators of HCC which appeared to be more evident when used in combination. This is probably due to the significant synergistic effect of the two inflammatory markers. Moreover, these markers correlated well with tumor response, as evidenced by serial measurements. The significant prognostic role of CRP and NLR is supported by the evidence that the levels of both markers displayed a linear relationship with the progressing stage of tumor and Child-Pugh classification, known as the two key prognostic factors for HCC.

There have been not yet clear explanation how elevated CRP and NLR would be responsible for tumor progression. Some studies have compiled the possible roles of CRP in cancer development as follows: i) anti-apoptotic activity and tumorigenic potency by over expression of IL-6, ii) T cell impairment, iii) resistance to chemotherapy, and iv) increased levels of serum angiogenic factors [23]. The levels of CRP have been inversely related to tumor-infiltrating CD4+ T-lymphocytes within the tumor microenvironment, which in turn carriers a poor prognosis [24]. Because not only normal hepatocytes but also hepatoma cells can produce serum CRP [25], it could be regarded that elevated CRP is related to hepatic tumor burden, and this has supporting evidence [7].

Although measured easily, NLR is more complicated due to its special feature as a combined factor of inflammation and host immune reaction. Increased counts of neutrophil can provide an adequate environment for tumor growth and even metastasis via angiogenesis. One study showed that neutrophils enhance tumor invasion via paracrine regulation mediated by neutrophil-derived hepatocyte growth factor [26]. The accumulated IL-17-recruited neutrophils into peritumoral stroma of HCC were the major source of matrix metalloproteinase-9, which stimulates proangiogenic activity in HCC [27]. Circulating vascular endothelial growth factor, a key proangiogenic factor, is contained in granulocyte, particularly in the neutrophils [28]. Neutrophil can contribute to cancer metastasis via promoting motility of cancer cells and adhesion to hepatic sinusoids [29,30]. Since neutrophil could also suppress T cell activation through the production of arginase, nitric oxide and reactive oxygen species [31], it induces depletion of lymphocyte-mediated immune response. Weaker immune reaction due to relative lymphocytopenia and elevated NLR could explain for depletion of tumor-infiltrating lymphocytes that are independently predictive of cancer-specific survival [32].

With multivariate analysis, the present study revealed Child-Pugh class, tumor size, tumor multiplicity, presence of PVT, AFP, CRP and NLR as independent factors predictive of outcome of HCC. A recent study evaluating CRP and NLR in transplant recipients with HCC showed that CRP did not affect overall patient survival, but NLR did with statistical significance [33]. The discrepancy could be due to some differences in assays measuring CRP and the study population. Our study employed a high-sensitivity CRP with cut-off value 6.3 mg/L, whereas the study detected CRP with a conventional sensitivity with cut-off value 1 mg/dL (= 10 mg/L). Additionally, our study recruited patients with various stages of HCC undergoing different treatment modalities, which is a distinction to the study recruiting patients with limited stages of HCC undergoing transplantation, which could result in no more lasting inflammation. There is no consensus on the cut-off value for NLR. It has been therefore set empirically between 2.42 and 5 in studies [5,14-20]. In this analysis, the cut-off value of 2.3 for NLR offered the most significant association with the patient outcomes. Further studies are needed to determine the optimal cut-off point of CRP and NLR in predicting prognosis in patients with HCC.

In our results, it is interesting to note that an elevated CRP level was strongly associated with an elevated NLR. It seems to be due to some relationship between these two markers in terms of host inflammation and immune reaction, as evidenced by the observations that high CRP levels associated with inflammation are inversely related to low-level tumor lymphocyte infiltration, which may contribute to an elevated NLR [24]. Although they had modest individual associations on the prognosis of the patients, the significance was quite stronger when used in combination. It is of note that both CRP and NLR levels were significantly related to tumor burden and underlying hepatic reserve, known as the two key determinants of HCC survival, showing a linear positive relationship with tumor stages or Child-Pugh classes. Thus, their role as prognostic indicators seems to be synergistically enhanced with combined use of them, as presented in our survival analysis.

Moreover, our results showed that patients with elevated CRP and NLR had worse treatment response, while those with a low-level of CRP and NLR had more favorable response. Consistent with survival analysis, there were significant differences in tumor response on the combined use of serum CRP level and NLR. Serial measurements of CRP and NLR exhibited a reduction in CRP and NLR among responders as well as their rise in progressive disease during treatment. Thus, it is expected that the outcome of treatment response before and during management would be predictable to some extent with the levels of CRP and NLR.

The current study has some limitations. There is some heterogeneity in treatment used for HCC, and the majority of the patients received TAC-based loco-regional therapy, with limited number of surgical cases, which could have affected the outcome. However, the prognostic effect of those inflammatory markers was still apparent when analyzed specifically in selected patients receiving loco-regional therapies. Although there are many other conditions affecting the levels of CRP and NLR, like an infectious state and autoimmune disease, the possibility may be minimized because most of those markers were indeed tested prior to treatment, without evidence of serious infection. Rather, the results of our study could be reliable, because we described the largest cohort to date of consecutive patients and provided comprehensive data from serial measurements of CRP and NLR simultaneously, which have not been explicitly evaluated thus far.

## Conclusions

In summary, this study demonstrated that CRP and NLR are not only markers of inflammation, but also independent prognostic indicators for HCC, reflecting tumor burden and hepatic reserve. Their role as a surrogate marker for tumor response and survival is more enhanced when used in combination. Further studies are needed to confirm and update a more detailed clinical relevance and biological mechanisms of CRP and NLR.

## Abbreviations

HCC: Hepatocellular carcinoma; HBV: Hepatitis B virus; HCV: Hepatitis C virus; CRP: C-reactive protein; IL: Interleukin; NLR: Neutrophil-to-lymphocyte ratio; PVT: Portal vein thrombosis; AFP: α-fetoprotein.

## Competing interests

The authors declare that they have no competing interests.

## Authors’ contributions

BSO and JWJ participated in the design of the study, performed the statistical analysis and interpretation of data, and drafted the manuscript. JHK, CRY and KWC recruited patients to the study. CSK is a radiation oncologist who contributed to treat the patients. HSJ is an interventional radiologist who contributed to treat the patients. SL is a clinical pathologist who participated in the study design. All authors read and approved the final manuscript.

## Authors’ information

Jeong Won Jang, Division of Hepatology, Department of Internal Medicine, College of Medicine, The Catholic University of Korea, #222 Banpo-daero 22-gil, Seocho-gu, Seoul 137-701, Korea, Telephone: +82-32-280-5866, Fax: +82-32-280-5987.

## Pre-publication history

The pre-publication history for this paper can be accessed here:

http://www.biomedcentral.com/1471-2407/13/78/prepub
